# PKC α regulates netrin-1/UNC5B-mediated survival pathway in bladder cancer

**DOI:** 10.1186/1471-2407-14-93

**Published:** 2014-02-15

**Authors:** Jiao Liu, Chui-ze Kong, Da-xin Gong, Zhe Zhang, Yu-yan Zhu

**Affiliations:** 1 Department of Urology, the First Affiliated Hospital of China Medical University, Shenyang, Liaoning 110001, China

**Keywords:** PKC α, Netrin-1, UNC5B, Bladder cancer

## Abstract

**Background:**

Netrin-1 and its receptor UNC5B play important roles in angiogenesis, embryonic development, cancer and inflammation. However, their expression patttern and biological roles in bladder cancer have not been well characterized. The present study aims to investigating the clinical significance of PKC α, netrin-1 and UNC5B in bladder cancer as well as their association with malignant biological behavior of cancer cells.

**Methods:**

Netrin-1 and UNC5B expression was examined in 120 bladder cancer specimens using immunohistochemistry and in 40 fresh cancer tissues by western blot. Immunofluorescence was performed in cancer cell lines. PKC α agonist PMA and PKC siRNA was employed in bladder cancer cells. CCK-8, wound healing assays and flow cytometry analysis were used to examine cell proliferation, migration and cell cycle, respectively.

**Results:**

Netrin-1 expression was positively correlated with histological grade, T stage, metastasis and poor prognosis in bladder cancer tissues. Immunofluorescence showed elevated netrin-1 and decreased UNC5B expression in bladder cancer cells compared with normal bladder cell line. Furthermore, cell proliferation, migration and cell cycle progression were promoted with PMA treatment while inhibited by calphostin C. In addition, PMA treatment could induce while calphostin C reduce netrin-1 expression in bladder cancer cells.

**Conclusions:**

The present study identified netrin-1/UNC5B, which could be regulated by PKC signaling, was important mediators of bladder cancer progression.

## Background

Bladder cancer (BC) is one of the most deadly urological malignant tumors and also the 2nd most common urologic cancer [1]. In the US, BC is the ninth most common cause of cancer-related mortality, and is the fourth most common cancer in men. Most bladder cancers are initially non-invasive and up to 15% will progress to muscle-invasive carcinoma. Although treatment of bladder cancer has been improved greatly, the mortality of this disease is still increasing [2].

As the central hub of a variety of signal transduction process, PKC involves in cell information transmission, secretion, cell differentiation and proliferation. What’s more, it participates in apoptosis and differentiation of tumor cells. PKC α is one subtype of classic protein kinase C, which is closely related to recurrence of bladder cancer [3]. PKC α can promote proliferation, migration and survival of cancer cells through the downstream signal transduction pathways ERK1/2 and NF-κB [4]. Recent research shows that activation, overexpression of PKC α as well as suppressing or depletion of PKC α can regulate the proliferation of cancer cells [5-7]. Thus it can be seen that PKC α is closely related to the biological behaviour of bladder cancer.

As a kind of proto-oncogene, Netrin-1 is the axon guidance factor that attracts the most attention in the family of dependence receptor [8]. Researches show that netrin-1 can activate PKC α after combination with its receptor, which may cause phosphorylation to promote cancer cell proliferation, and then restrain cell proliferation [9]. In recent years, netrin-1 has been found effective in inhibiting apoptosis in lung cancer, advanced neuroblastoma, breast cancer and prostate cancer [10-13]. UNC5B is one of the dependence receptors of netrin-1. Researches show that UNC5B is the downstream gene of p53, down-regulation of UNC5B using small interfering RNA Can significantly inhibit apoptosis, thus concludes that UNC5B plays a role of inducing apoptosis, and it is a kind of tumor suppressor genes [14]. According to reports in the literature, up-regulation of netrin-1transcripts can antagonize apoptosis induced by UNC5B [15]. Since PKC α, netrin-1and UNC5B play a significant role in the process of tumor treatment. Therefore, study the mechanisms of action of PKC alpha regulates netrin-1/UNC5B-mediated survival pathway is of great significance.

In this study, we detect the expression of netrin-1/UNC5B in the bladder cancer tissues as well as in the bladder cancer cell line on both the RNA and protein levels, we found that netrin-1/UNC5B was closely related to the activation of PKC alpha state. Furthermore, netrin-1/UNC5B was closely associated with bladder cancer malignant pathological biological behavior. Therefore, we need to validate that PKC α inhibits bladder cancer cell apoptosis by regulating signaling pathway of netrin-1/UNC5B.

## Methods

### Patients and specimens

One hundred and twenty bladder cancer tissues were collected by the surgical resection in the First Affiliated Hospital of China Medical University from 2008 to 2012. Bladder cancer tissues and paired adjacent normal bladder tissues were collected. None of patients underwent chemotherapy, radiotherapy or adjuvant treatment before surgery. Patients’ consent for the research use of tumor tissue was obtained, and the research protocol was approved by Ethical Committee at China Medical University. We followed up all patients for the survival time by consulting their case documents and telephoning.

### Cell culture, treatment of cells with drugs and siRNA

Human BC cell lines SV, 5637, T24 and BIU-87 were purchased from Cell Bank of Shanghai Institutes for Biological Sciences, Chinese Academy of Sciences. They were maintained in RPMI 1640, or DMEM medium supplemented with 10% fetal bovine serum (FBS). Cells were incubated at 37°C in 5% CO_2_.

For PMA treatment, cells were treated at the concentration of 100 nmol/L for 24 hours. For calphostin C treatment, cells were treated by using 100 nmol/L PMA for 4 hours first, then 50 nmol/L calphostin C for 24 hours. For siRNA transfection, Lipofectamine (Invitrogen) was used. PKC siRNA sequences was as follows: forward, 5’ GUG CCA UGA AUU UGU UAC UTT 3’, reverse, 5’ AGU AAC AAA UUC AUG GCA CTT 3’.

### Real-time PCR

Total cellular RNA was extracted from cells using the RNeasy Plus Mini Kit (Qiagen). First strand of cDNA was synthesized by using PrimeScript RT reagent kit (Takara). Quantitative real-time polymerase chain reaction (QPCR) was done using SYBR Green PCR Master Mix (Applied Biosystems) in a total volume of 20 μl on a 7900 Real-Time PCR System (Applied Biosystems): 50°C for 2 min, 95°C for 5 min, 45 cycles of 95°C for 40 s, 60°C for 30 s. The sequences of the primer pairs are: UNC5B forward, 5’ CAG GGC AAG TTC TAC GAG AT 3’, reverse, 5’ TGG TCC AGC AGG ATG TGA 3’, netrin-1 forward, 5’ GTC AAT GCG GCC TTC GG 3’, reverse, 5’ CTG CTC GTT CTG CTT GGT GAT 3’, *β*-actin forward, 5’ TTA GTT GCG TTA CAC CCT TTC 3’, reverse, 5’ ACC TTC ACC GTT CCA GTT T 3’, *β*-actin was used as the reference gene. Relative gene expression levels were represented as ΔCT = CT gene – CT reference; fold change of gene expression was computed by the 2^−ΔΔCT^ method [16]. Experiments were repeated in triplicate.

### Western blotting

Total protein from cells was extracted in lysis buffer (Pierce) and quantified using the Bradford method. Total protein was separated by SDS-PAGE (12%). After transferring to polyvinylidene fluoride (PVDF) membrane (Millipore, Billerica, MA), the membranes were incubated overnight at 4°C with antibodies against UNC5B/netrin-1 (1:1000, Abcam Inc. USA), GAPDH (1:500, Santa Cruz Biotechnology). After incubation with peroxidase-coupled anti-mouse/rabbit IgG (Santa Cruz Biotechnology) at 37°C for 2 h, bound proteins were visualized using ECL (Pierce) and detected using BioImaging Systems (UVP Inc., Upland, CA). The relative protein levels were calculated based on GAPDH protein as a loading control.

### Immunohistochemistry and evaluation

Sections were deparaffinized in xylene, hydrated in graded alcohols. After antigen retrieval, sections were incubated in an aqueous solution of 3% hydrogen peroxide followed by incubation with 5% non-fat milk, which served as a blocking agent for nonspecific binding. Slides were incubated with UNC5B & netrin-1 rabbit polyclonal antibody with an optimal dilution of 1:100 overnight at 4°C. Biotinylated goat anti-rabbit serum IgG was used as a secondary antibody. After washing, the sections were incubated with streptavidin–biotin conjugated with horseradish peroxidase at room temperature for 10 min, and the peroxidase reaction was developed with 3, 3′-diaminobenzidine tetrahydrochloride. All the slides were evaluated by 2 pathologists. Five views were examined per slide; 100 cells were observed per view at × 400 magnification. Nucleus and/or cytoplasmic immune-staining in tumor cells were considered positively. Positive reactions were scored for both intensity of staining and percentage of positive cells. Intensity grades were 0 (no staining), 1 (weak, light yellow), 2(moderate, yellowish brown), to 3 (intense, brown) and the percentage of positive tumor cells were scored as 0 (negative), 1 (1–50%), 2 (51–75%), 3 (≥76%). Scores of each sample were multiplied to give final scores of 0–9, and the tumors were finally determined as negative: score 0; low expression: 0 < score ≤ 4; or high expression: score > 4.

### Cell proliferation and invasion assays

Cell Counting Kit-8 (Dojindo) was employed to determine the number of viable BIU cells. Experiments were performed according the manufacturer’s protocol. Invasion ability was examined by wound healing assay. In brief, cells were seeded at a density of 1.0 × 10^6^ cells/well in 6-well culture plates. After they grown into confluence, scratch was performed using a pipette tip, cells were washed with PBS and cultured in the FBF-free medium for 24 hours and photographed.

### Cell cycle by flow cytometry

Cells with different treatment were harvested, fixed in 1% paraformaldehyde, washed with phosphate-buffered saline (PBS), and stained in 5 mg/ml propidium iodide in PBS supplemented with RNase A (Roche, Indianapolis, IN) for 30 minutes at room temperature. Data were collected using BD systems.

### Immunofluorescence

Cells were washed with PBS, fixed with 4% formaldehyde, permeabilized with 0.2% Triton X-100 at 37°C, and incubated in 5% BSA. Then cells were incubated with rabbit anti-human netrin-1 & UNC5B antibody (1:100) and mouse anti-human PKC α antibody (1:50) overnight at 4°C. Then fluorescently labeled goat anti-rabbit IgG (1:200) were added at 37°C for 1 h. Nucleus was stained with DAPI. Cells was then observed using fluorescence microscope.

### Statistical analysis

SPSS 13.0(SPSS Inc, Chicago, IL) was used for statistical analysis. The χ^2^ test was used to evaluate the association between the expression of netrin-1 & UNC5B and clinicopathologic variables. Kaplan-Meier method and log-rank test were used for survival analysis. The t-test was used to analyze the difference for western blot data. p values < 0.05 was considered significant.

## Results

### Expression of netrin-1 and UNC5B in bladder cancer tissues and association between their expressions & clinicopathologic parameters

**Figure 1:**
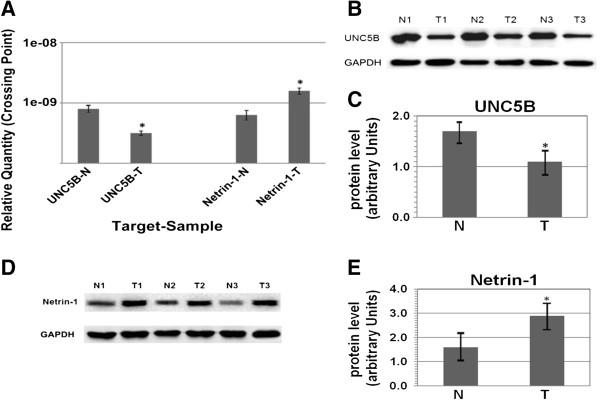
**Expression by RT-PCR and western blot in BC tissues (T) and normal bladder tissues (N). (A)** The average netrin-1 & UNC5B expression ± SD for all studied tumors (120 cases) and their corresponding normal tissues (40 cases) by RT-PCR, bar graphs describe significant UNC5B down-regulation and netrin-1 up-regulation in T in comparison with N (*P* < 0.05), β-actin as an internal control. **(B)** UNC5B protein was detected by western blot in 40 pair tissues and band intensities indicate UNC5B expression conspicuously lower in T comparing with N. GAPDH was used as a loading control. **(C)** The ratio between the optical densities of UNC5B & GAPDH in the same tissue was calculated and expressed graphically. Significant differences of UNC5B expression between T & N were analyzed statistically and UNC5B expression was obviously greater in N (*P* < 0.05). **(D)** Netrin-1 protein was detected by western blot and increased in T. **(E)** The relative protein expression of netrin-1, GAPDH as an internal control (*P* < 0.05).

**Figure 2:**
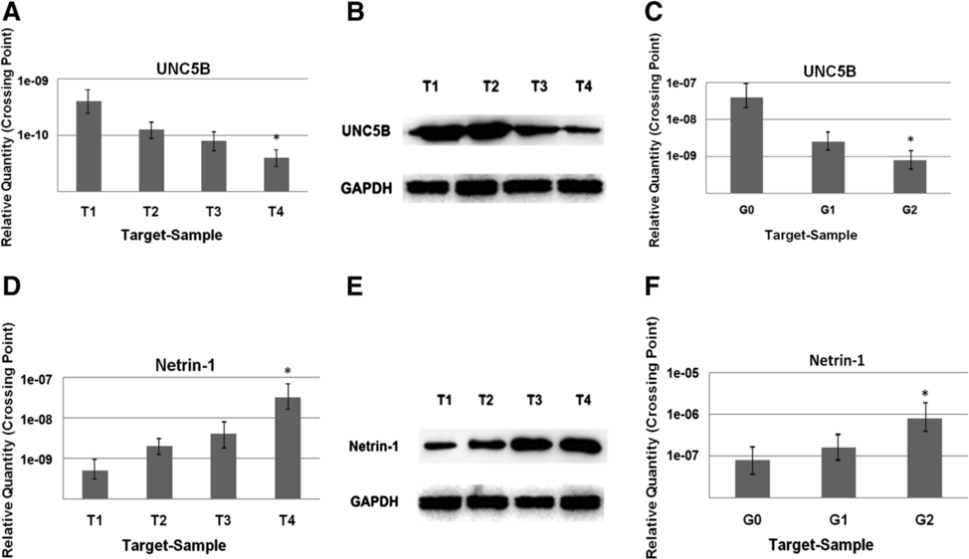
**Expressions by RT-PCR and western blot in different stages and grades. (A)** UNC5B expression was different in BC specimens with different clinical pathological stages (T1, T2, T3 & T4) at mRNA level. Data was analyzed by Student’s *t* test and shown as mean ± SD of ten independent experiments. UNC5B expression in stage T4 was much less than in T1–T3 stages (*P* < 0.05). *P < 0.05 compared with stage T1 BC tissues (paired *t*-test). **(B)** Different expressions of UNC5B in *pT* stage were detected by western blot and UNC5B expression in stage T4 was sharply less than in T1–T3 stages (*P* < 0.05). **(C)** UNC5B mRNA expression was determined by RT-PCR in different grades (G1, G2 & G3). Differences at G1 & G3 were significant. *P < 0.05 compared with G1 BC tissues (paired *t*-test). **(D)** Netrin-1 expression was different in BC specimens with different *pT* stage at mRNA level by RT-PCR. Stage T4 was more than in T1–T3 stages (*P* < 0.05). **(E)** Western blot was used to detect netrin-1 expression in different stages. The band intensities indicate netrin-1 expression more in stage T4. **(F)** Netrin-1 expression was determined by RT-PCR in different grades (G1, G2 & G3), and G3 was the highest (*P* < 0.05). *P < 0.05compared with G1 BC tissues (paired *t*-test). All data are representative of 3 individual experiments.

Quantitative real-time PCR (RT-PCR) and western blot analysis were used to evaluate netrin-1 & UNC5B expression in 120 BC tissues and 40 normal bladder epithelial tissues. It showed that the increased netrin-1 expression and decreased UNC5B expression could be detected in BC samples compared with the normal bladder samples (*P* < 0.05). The mRNA expression of netrin-1 was found to be increased, while that of UNC5B decreased in the BC tissues as compared with the normal bladder epithelial tissues. The protein expression of netrin-1 and UNC5B showed the same trend as that of mRNA expression, and the optical density of all tumor (T) & normal (N) tissues were measured and expressed graphically (Figure 1). Differences of mRNA expression and protein levels in different *T* stage (T1, T2, T3 & T4) and histological grade (G1, G2 & G3) were significant (*P* < 0.05) (Figure 2).

**Figure 3:**
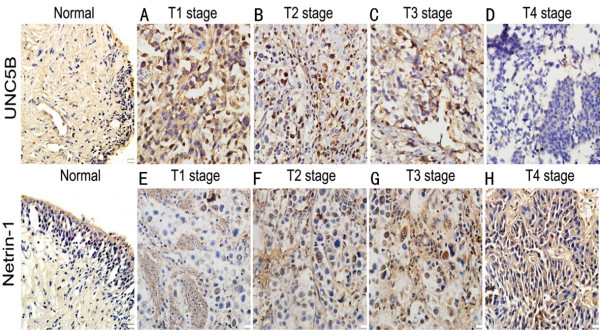
**Representative images from immunohistochemical staining in different histological stages UNC5B was localized at cytoplasm and netrin-1 was mainly in cell nucleus and partly in cell cytoplasm of tumor tissues with granular brown staining.** Almost all T1 tumor tissues showed strong UNC5B staining intensity. UNC5B expression in stage T4 was significantly less than in stage T1–T3 specimens (Figure 3**A**, **B**, **C & D**) (*P* = 0.021). While netrin-1 showed strong expression in stage T4 than other stages (T3, T2 & T1) and was associated significantly with *pT* stage (Figure 3**E**, **F**, **G & H**) (*P* = 0.013). Representative tissue sections with different immunointensity of UNC5B protein in A T1, B T2, C T3 & D T4. E Low level of netrin-1 expression in T1, H High level of netrin-1 expression in T4, F & G represent T2 &T3. Magnification × 400. *P < 0.05 compared with stage normal bladder tissues (paired *t*-test).

The expression of netrin-1 protein in BC and normal adjacent tissues was located in both cytoplasm and nucleus, while UNC5B protein appeared to be located only in cytoplasm (Figure 3). Elevated expression of netrin-1 and down-regulated level of UNC5B was observed in T4 tumors compared with normal adjacent tissues (*P* < 0.01). To explore the relationship of netrin-1 over-expression and UNC5B down-regulation in a large cohort of BCs, we examined the correlation between the immunostaining of netrin-1 & UNC5B and clinic-pathological features including age, gender, tumor size, tumor grade, etc. There was a statistically significant positive correlation between UNC5B & netrin-1 expression and high grade, aggressive stage and metastasis (Tables 1 and 2), the expression of UNC5B was finally determined T/N < 0.5 as low expression & T/N > = 0.5 as normal expression and the expression of netrin-1 was considered T/N > 2 as high expression & T/N < = 2 as normal expression.

**Table 1:** Relationship between the expression of UNC5B and clinicopathologic factors in BC patients

**Factors**	**Group**	**UNC5B expression**		**X**^**2**^	***P *** **value**
		**High(%)**	**Low(%)**		
Gender	Male	52(43.3%)	25(20.8%)	0.029	0.162
	female	29(24.2%)	14(11.7%)		
Age at surgery	<55	29(24.2%)	18(15.0%)	2.152	0.247
	≥55	44(36.6%)	29(24.2%)		
Histologic grade	G1	18(15.0%))	5(4.2%)	7.537	0.032
	G2	49(40.8%)	15(12.5%)		
	G3	17(14.2%)	16(13.3%)		
PT Stage	T1	29(24.2%)	6(5.0%)	19.564	0.014
	T2	23(19.2%)	12(10.0%)		
	T3	13(10.8%)	12(10.0%)		
	T4	6(5.0%)	19(15.8%)		
Tumor size(mm)	<10	16(13.3%)	5(4.2%)	2.011	0.436
	10-30	32(26.7%)	24(20.0%)		
	>30	18(15.0%)	25(20.8%)		
Multiplicity	Unifocal	28(23.3%)	24(20.0%)	1.593	0.312
	Multifocal	39(32.5%)	29(24.2%)		
Recurrence	Yes	39(32.5%)	43(35.8%)	31.573	0.220
	No	14(11.7%)	24(20.0%)		
Metastasis	Yes	7(5.8%)	19(15.8%)	13.253	0.001
	No	69(57.5%)	25(20.9%)		

**Table 2:** Relationship between the expression of netrin-1 and clinicopathologic factors in BC patients

**Factors**	**Group**	**Netrin-1 expression**		**X**^**2**^	***P *** **value**
		**High(%)**	**Low(%)**		
Gender	Male	49(40.9%)	28(23.3%)	0.037	0.213
	female	28(23.3%)	15(12.5%)		
Age at surgery	<55	24(20.0%)	23(19.2%)	1.984	0.198
	≥55	25(20.8%)	48(40.0%)		
Histologic grade	G1	3(2.5%)	20(16.7%)	7.632	0.024
	G2	38(31.6%)	26(21.7%)		
	G3	29(24.2%)	4(3.3%)		
PT Stage	T1	7(5.8%)	28(23.3%)	21.135	0.031
	T2	11(9.2%)	24(20.0%)		
	T3	16(13.4%)	9(7.5%)		
	T4	21(17.5%)	4(3.3%)		
Tumor size(mm)	<10	5(4.2%)	16(13.3%)	1.794	0.153
	10-30	32(26.7%)	24(20.0%)		
	>30	24(20.0%)	19(15.8%)		
Multiplicity	Unifocal	26(21.7%)	26(21.7%)	1.782	0.264
	Multifocal	32(26.6%)	36(30.0%)		
Recurrence	Yes	52(43.3%)	30(25.0%)	32.236	0.658
	No	24(20.0%)	14(11.7%)		
Metastasis	Yes	21(17.5%)	5(4.2%)	10.896	0.002
	No	9(7.5%)	85(70.8%)		

**Figure 4:**
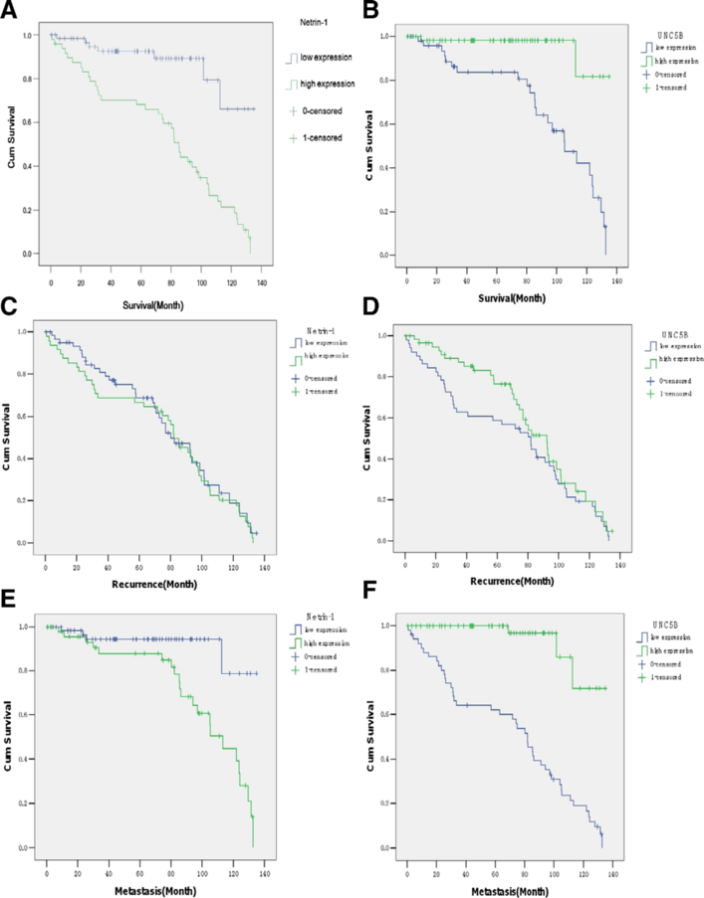
**Survival, recurrence and metastasis curve analysis by the Kaplan-Meier method. (A)** Patients with intense netrin-1 expression had significantly shorter median survival time (76.624 months) than those with weak netrin-1 expression (117.981 months) through log-rank univariate analysis (n = 120, *P* < 0.01). **(B)** Patients with weak UNC5B expression had significantly shorter median survival time (96.881 months) than those with intense UNC5B expression (128.939 months) (*P* < 0.01). **(C)** Recurrence curve analysis indicated that the difference was not statistically significant with intense netrin-1 expression (74.463 months) and lower expression (79.505 months). (*P* > 0.01) **(D)** The difference was not statistically significant in more (84.47 months) and less (69.225 months) UNC5B expression by recurrence curve analysis. (*P* > 0.01) **(E)** Patients with intense netrin-1 expression had significantly shorter median metastasis time (100.836 months) than those with weak netrin-1 expression (124.946 months). (*P* < 0.01) **(F)** Patients with weak UNC5B expression had significantly shorter median metastasis time (71.243 months) than those with intense UNC5B expression (125.957 months). (*P* < 0.01)

During follow-up period, 70.0% (21 of 30) of tumors with high netrin-1 expression developed metastasis compared with 5.6% (5 of 90) of tumors with low netrin-1 expression, (*P* < 0.01). Meanwhile, 43.2% (19 of 44) of tumors with low UNC5B expression showed metastasis, compared with only 9.2% (7 of 76) of tumors with high UNC5B expression having metastasis (*P* < 0.01). Therefore, high expression of netrin-1 and low expression of UNC5B were positively associated with metastasis of BC. Kaplan-Meier plots and log-rank tests showed that patients with high netrin-1 expression and low UNC5B expression in their tumor tissues had statistically significant shorter survival rate compared with those with low netrin-1 expression and high UNC5B expression (*P* < 0.01). However, there was no significant association between tumor recurrence and intense & feeble netrin-1 expression; recurrence curve analysis also indicated that the difference was not statistically significant with high & low UNC5B expression (*P* > 0.01). Moreover, we found that patients with high netrin-1 expression and low UNC5B expression had statistically significant higher metastasis rate compared with those with low netrin-1 expression and high UNC5B expression (*P* < 0.01; Figure 4). Log-rank analysis also showed that the expression of netrin-1 & UNC5B (*P* < 0.01) were significant predictors of the metastasis of BC and had statistically significant independent association with poor prognosis of the patients.

### Netrin-1 & UNC5B expression and location in BC cell lines

**Figure 5:**
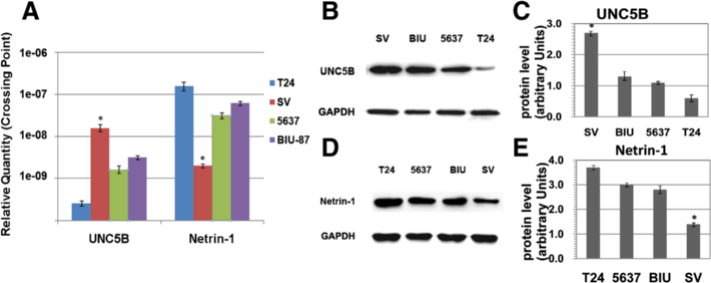
**Expressions by RT-PCR and western blot in bladder cell lines. (A)** Netrin-1 & UNC5B mRNA expression in four bladder cell lines (T24, SV, 5637, and BIU-87) was assayed using quantitative real-time PCR. To normalize Netrin-1 & UNC5B expression, *β*-actin was used as internal control. Each expression level was shown as mean + SD. UNC5B expression was significantly greater in SV cells than in 5637, T24 or BIU-87, while netrin-1 was lower in SV than in BC cell lines. *P < 0.05 compared with BC cell lines including 5637, T24 and BIU-87 (paired *t-test*). **(B)** UNC5B &**(D)** Netrin-1 expression at protein level in different cell lines (SV, BIU, 5637 and T24) were analyzed by western blots with 50 μg protein extracts. GAPDH was selected to be endogenous control. The differences in UNC5B expression between 5637, T24 and BIU-87 were significant. SV cells represent bladder normal cell lines of which UNC5B expression is the most, the others are BC cell lines, and netrin-1 expresses the least in SV. **(C) & (E)** The ratio between the optical density of UNC5B & netrin-1 and GAPDH of the same tissue was calculated and expressed graphically (*P* < 0.05). *P < 0.05 compared with BC cell lines (paired *t*-test).

**Figure 6:**
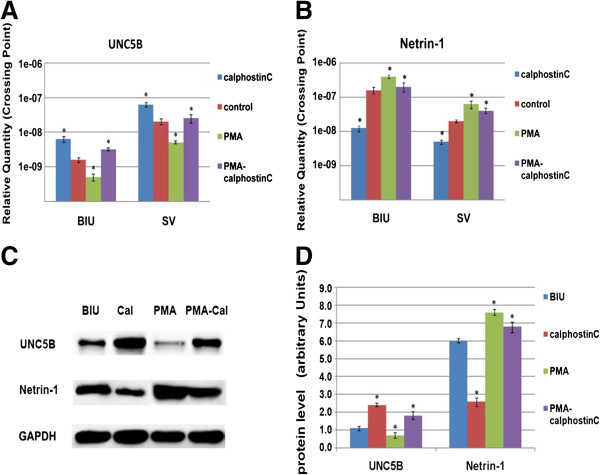
**Expressions by RT-PCR and western blot in bladder cells with drugs. (A) & (B)** Quantitative real-time PCR (RT-PCR) were used to evaluate netrin-1 & UNC5B’s expression in treatment of cells (BIU & SV) with drugs- PMA (PKC α agonist) and calphostin C (PKC α inhibitor). The results showed that the cells were significantly inhibited by calphostin C (24 h) in netrin-1’s expression and promoted by PMA (24 h), and cells were first promoted, and then inhibited by PMA (4 h) then calphostin C (24 h). Whlie UNC5B was opposite. **(C)** Confirmation of netrin-1 down-regulation with calphostin C and up-regulation with PMA by western blot. **(D)** The relative protein expression of netrin-1 & UNC5B in treatment of cells with drugs, GAPDH as an internal control (*P* < 0.05). *P < 0.05 compared with BC cell line BIU (paired *t*-test).

**Figure 7:**
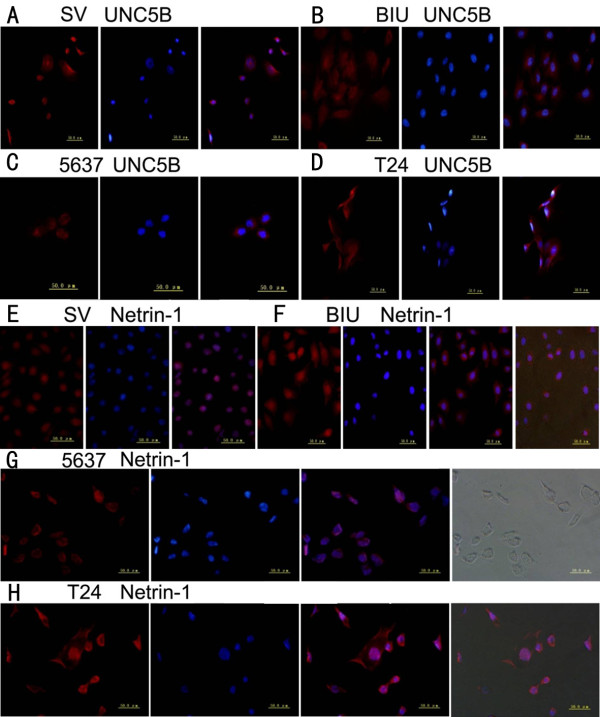
**Detection of UNC5B expression by immunefluorescence in four cell lines (SV, BIU, 5637 & T24) (A & E) SV, (B & F) BIU, (C & G) 5637, (D & H) T24. (A, B, C & D)** The expression of UNC5B located in cytoplasm (red color). The blue ones were cell nucleolus. Other ones are transmission light figures. From these figures we can see cells’ form clearly. UNC5B location was almost the same in SV, 5637, T24 & BIU-87 bladder cancer cells lines. **(E, F, G & H)** The localization in four cell lines (SV, BIU, 5637 & T24) of netrin-1 was found by immunofluorescence mainly in cell nucleus and partly in cell cytoplasm.

Quantitative real-time PCR and western blot analysis were used to evaluate the expression of netrin-1 & UNC5B in human bladder cell lines SV, BIU-87, 5637 & T24, and immunofluorescence was used to detect of netrin-1 & UNC5B expression and localization. The results showed that highly invasive BC T24 cells had stronger netrin-1 expression than the superficial BC BIU-87 & 5637 cells and normal SV cells which had lowest expression. Opposite trend was observed regarding UNC5B expression (Figure 5), It was further confirmed that the expression of netrin-1 & UNC5B was positively correlated with BC grade. Quantitative real-time PCR and western blot analysis were also used to evaluate netrin-1 & UNC5B’s expression after PMA (PKC α agonist) and calphostin C (PKC α inhibitor) treatment. The results showed that netrin-1 expression were significantly inhibited by calphostin C and enhanced by PMA (treat for 24 h), while UNC5B showed the opposite trend (Figure 6). Immunofluorescence results showed that UNC5B was expressed in BC cell cytoplasm in all these four cell lines, while netrin-1 was found mainly located in cell nucleus and partly in cell cytoplasm (Figure 7).

### BC cells treated with PKC α agonist & inhibitor & siRNA

**Figure 8:**
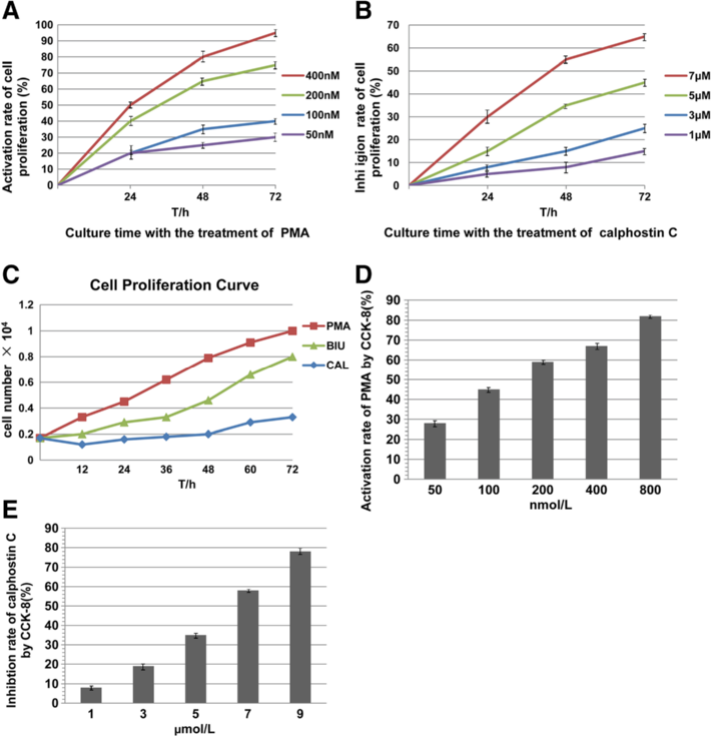
**Proliferation and invasion ability of BIU were inhibited by calphostin C and promoted by PMA. (A)** CCK-8 assay was used to examine BIU cell proliferation. cell proliferation was inhibited on dose-dependent correlation with the increasing concentration (1, 3, 5, 7 μmol/L) of calphostin C at 24 h, 48 h, 72 h. **(B)** Cell proliferation was promoted by PMA on correlation with the increasing concentration (50, 100, 200, 400 nmol/L) at 24 h, 48 h, 72 h. **(C)** According A450 of BIU at different times, cell proliferation curve was drawn. Compared with BIU, cells with calphostin C were inhibited and cells with PMA were promoted. **(D) & (E)** According the optical density value **(A)** of each well measured, BIU cell growth inhibition/activation ratio was calculated as (1 – A_450_ of experimental well/A_450_ of blank control well) × 100%, with different concentration of PMA (50, 100, 200, 400 nmol/L) and calphostin C (1, 3, 5, 7 μmol/L), we calculated the activation/inhibition ratio. Each data are representative of 3 individual experiments. IC50 of calphostin C =7.4 μmol/L, and IC50 of PMA = 24 nmol/L.

**Figure 9:**
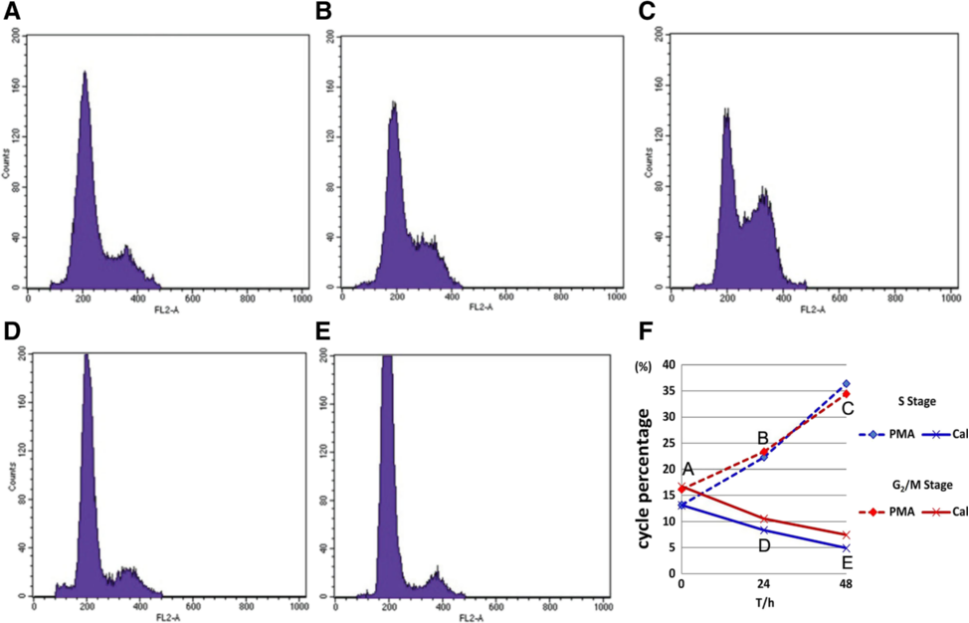
**Detection of cell cycle by flow cytometry. (A)** Flow cytometry was used to examine BIU cell cycle. G1 / G2, S, G2/M phase occupied 57.19%, 13.14%, 16.72%, respectively. **(B) & (C)** With the increasing treatment time (24 h, 48 h) of PMA, the number of BIU cells increased gradually in S phase and G2/M phase, and S phase was 22.33% for 24 h, 36.41% for 48 h; G2/M phase was 23.39% for 24 h, 34.42% for 48 h. **(D) & (E)** With the increasing treatment time (24 h, 48 h) of calphostin C, the number of BIU cells decreased noticeably in S phase and G2/M phase, and S phase was 8.39% for 24 h, 4.92% for 48 h; G2/M phase was 10.55% for 24 h, 7.46% for 48 h. The data are representative of 3 individual experiments. **(F)** Summary of cell cycle distribution in panel **A**-**E**.

PKC α inhibitor and agonist, PMA & calphostin C, were applied to treat BC BIU cells. We found that cell proliferative and invasive activities were significantly increased after PMA treatment, but decreased by calphostin C treatment (Figure 8). Moreover, the FCS showed that cell cycle was accelerated by PMA treatment (S phase 22.33% for 24 h, 36.41% for 48 h; G2/M phase 23.39% for 24 h, 34.42% for 48 h) and blocked by calphostin C treatment at both S phase and G2/M phase (S phase 8.39% for 24 h, 4.92% for 48 h; G2/M phase 10.55% for 24 h, 7.46% for 48 h ) compared with BIU cell without drugs (S phase 13.14%; G2/M phase 16.72%) (Figure 9); the cells mainly concentrated in G1/G2 phase (almost the same percentage at 57.19%) from mitotic completion to DNA replication.

**Figure 10:**
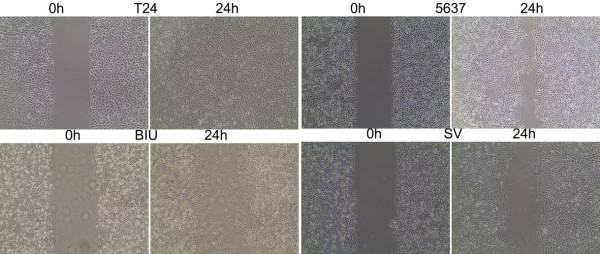
**Migration and invasion assays were used by wound healing in BC cells (SV, 5637, BIU, T24).** Comparing scratches width in order to verify the invasion capability of the prostate carcinoma cells. Results showed that the most invasive cells were T24 and all BC cells were invasive.

**Figure 11:**
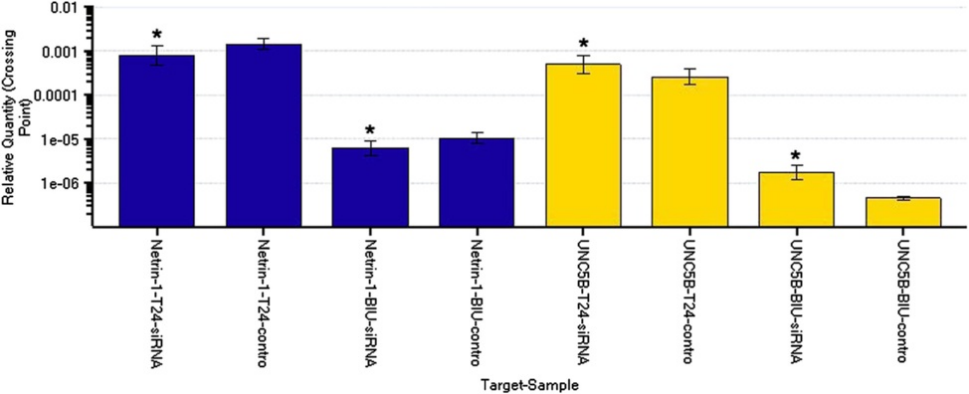
**PKC siRNAs were transfected into bladder cancer cells T24 & BIU-87 transiently.** Real-time PCR results found after transfection PKC siRNA, netrin-1 was inhibited and the expression was decreased, while UNC5B was activated and the expression was elevated.

**Figure 12:**
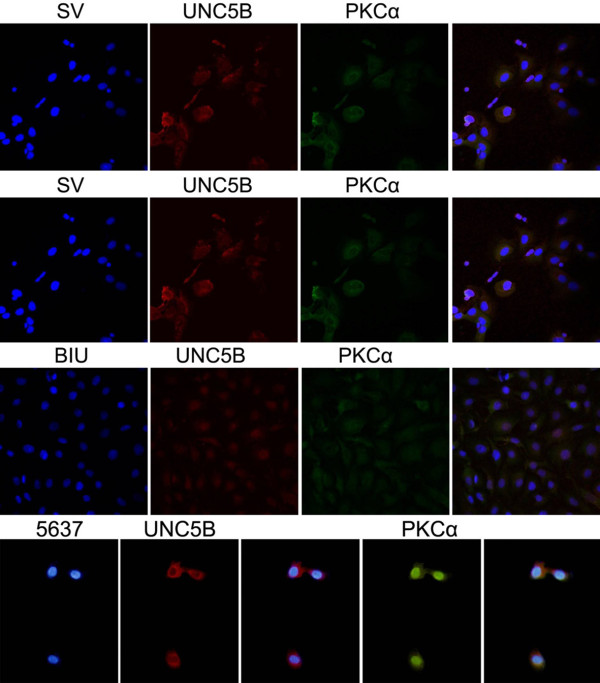
**The confocal immunofluorescence displayed that the existence of co-localization expression of PKC α and UNC5B.** Results showed that PKC α (green) and UNC5B (red) were observed and a co-localization expression of PKC α and UNC5B could be observed (yellow). Nuclear staining was performed with DAPI (blue) in panels.

Migration of bladder cancer cells by wound healing. Cells were seeded at 1.0 × 10^6^ cells/well in 6-well plates. After grown to confluence, the cell monolayer in each well was scraped with a pipette tip to create a scratch. Cells were washed by PBS three times and cultured in the FBS-free medium. Cells were photographed after 24 h and the scratch area was measured using Image software (Figure 10). PKC siRNAs were transfected into bladder cancer cells T24 & BIU-87 transiently. Real-time PCR showed that, netrin-1 expression was elevated after transfection with PKC siRNA, while UNC5B expression was decreased (Figure 11). The immunofluorescence confirmed the co-localization of PKC α and UNC5B, suggesting that the presence of their endogenous binding (Figure 12).

## Discussion and conclusions

Protein Kinase C (PKC), as the hub of a variety of signal transduction process, is not only involved in cell communication, secretion, cell differentiation & proliferation, but more importantly involved in tumor cell apoptosis and differentiation. PKC α is a classical Protein of Kinase C isoforms. Our and others’ research have shown that PKC α of high activation status is closely related to activation and apoptosis of bladder cancer recurrence [3]. UNC5B is abnormally expressed and associated with a highly malignant, chemotherapy-related and poor prognosis in colon cancer. It was reported that netrin-1 binding to its receptor can activate PKC α and lead to tumor cell proliferation, but it did not clarify PKC α and netrin-1/UNC5B’s regulatory mechanisms. To this end, we explored the mechanism of PKC α with netrin-1/UNC5B in bladder cancer. Our work shows that, PKC α, netrin-1 & UNC5B is closely related to the degree of malignancy and progress in bladder cancer and found PKC α promoted the survival of bladder cancer cell potentially through netrin-1/UNC5B signaling pathway. Thus, PKC α has an important influence on netrin-1/UNC5B signaling pathway & bladder cancer’s occurrence and development.

The expressions of netrin-1/UNC5B were detected in bladder cancer tissues & adjacent tissues and the relevance and the relationship with clinic pathological parameters was analyzed. The results showed that UNC5B had higher expression in adjacent tissues than bladder cancer tissues and it had higher expression in the low-level cancer tissues than in high-level ones, but netrin-1 in the opposite. According to immunohistochemical results, it showed UNC5B expression in the cytoplasm and netrin-1 existing in the cytoplasm and nucleus; netrin-1’s expression gradually increased from the bladder mucosa - transitional cell carcinoma and high - grade cancer evolution, while UNC5B is gradually reduced; netrin-1/UNC5B high/low expression is closely related to bladder cancer clinical grading, staging & metastasis; and Pearson correlation analysis showed that netrin-1 and UNC5B are negatively correlated. Netrin-1/UNC5B’s expression is proved to exist in kidney cancer and prostate cancer [13,17], and found that netrin-1 inhibits apoptosis in lung cancer, with advanced neuroblastoma, breast cancer [10-12]; UNC5B is one of the dependent receptors of netrin-1, and previous studies had demonstrated that increasing netrin-1 transcription can antagonize UNC5B induced apoptosis [15], which is consistent with the results of this study. Previously we have confirmed that PKC α is closely related to bladder cancer cell’s apoptosis & recurrence [3], and that netrin-1’s binding to its receptor UNC5B can cause PKC α phosphorylation and promote cancer cell proliferation [9], but it had not been confirmed in bladder cancer, for which we had done further research.

From the cellular level, it revealed netrin-1/UNC5B’s expression & location in bladder carcinoma. Four kinds of bladder cancer cell line T24, BIU-87, 5637 and SV malignancy has been clearly stated in previous studies: BIU-87, 5637, T24 are all bladder carcinoma cells, and their degree of malignancy increased in turn, and SV-HUC-1 is normal urothelial line [18]. We detected netrin-1/UNC5B expression in bladder cancer cell line from RNA and protein levels by Real-time PCR & Western-blot ion, UNC5B’ expression was the highest in normal bladder cell line (SV), and the expression was the lowest in the most malignant cells of T24, netrin-1 was the opposite. Immunofluorescence results showed that UNC5B was in bladder cancer pulp while expressions of netrin-1 existed in the cytoplasm and the nucleus. Netrin-1/UNC5B’s expression in cells and tissues shows consistent trend, and are related with the degree of malignancy of bladder cancer cell lines. PKC α has been shown to be involved in tumor cell apoptosis and differentiation. The high expression of PKC α in bladder cancer cells was found to promote cancer cell proliferation, and inhibit apoptosis and differentiation [3].

When bladder cancer cell was given PKC inhibitors and activators, and detected changes of netrin-1/UNC5B expression and bladder cell cycle, proliferation and apoptosis; it can be further confirmed that netrin-1/UNC5B are closely related with PKC α activation. When bladder cancer cell BIU-87 was given PKC inhibitors (calphoatin C) and activators (PMA), Real-time PCR & Western-blot showed that netrin-1 was inhibited after inhibitor treatment, while UNC5B was activated; netrin-1 was activated after PMA treatment, while UNC5B was suppressed. When CCK-8 and flow cytometry detection was carried out after drug treatment on bladder cancer cycle, proliferation and apoptosis. CCK-8 was found in best status by calphoatin C or PMA for 48 hours, and the inhibition rate & the activation rate increased with the increasing concentration, and at the same time it can be drawn that calphostin C of IC50 = 7.4 μmol/L, PMA’s IC50 = 24 nmol/L. Flow cytometry showed S and G2/M were inhibited or activated after calphoatin C or PMA treatment BIU in better condition after 48 hours. These results could confirm that netrin-1/UNC5B was closely associated with PKC α activation, and PKC α activation or inhibition might affect the proliferation and survival of cancer cells [4,6,7].

After transiently transfecting PKC siRNA into the bladder cancer T24 and BIU-87 cells, it clarified PKC α’s regulatory mechanismson on netrin-1/UNC5B; Real-time PCR test results showed that netrin-1 was inhibited after PKC siRNA transfection, with its expression decreased, while UNC5B increased. Immunofluorescence results revealed the presence of co-localization of PKC α with UNC5B expression. So we speculate that there may be endogenous binding.

From the above results, we can conclude that: PKC α can promote bladder cancer cell proliferation through the regulation of netrin-1/UNC5B. On this basis, we can intervene any stage in which PKC α and netrin-1/UNC5B affect, so as to control the proliferation of bladder cancer, and provide adequate theoretical basis for bladder cancer’s diagnosis and treatment.

## Competing interest

The authors declare that they have no competing interests.

## Authors’ contributions

The study was conceived by JL, DG, YZ and CK. Experiments were carried out by JL, ZZ and CK. Statistical analysis was carried out by JL. Manuscript was written by JL. All authors read and approved the final manuscript.

## Pre-publication history

The pre-publication history for this paper can be accessed here:

http://www.biomedcentral.com/1471-2407/14/93/prepub
